# Supplementary material for: The effects of queen mandibular pheromone on nurse-aged honey bee (Apis mellifera) hypopharyngeal gland size and lipid metabolism
Source: PLoS One. 2024 Sep 6;19(9):e0292500. doi: 10.1371/journal.pone.0292500 (PMC11379314; doi:10.1371/journal.pone.0292500)
Supplement: S1 Fig — (DOCX) [file pone.0292500.s001.docx]

**
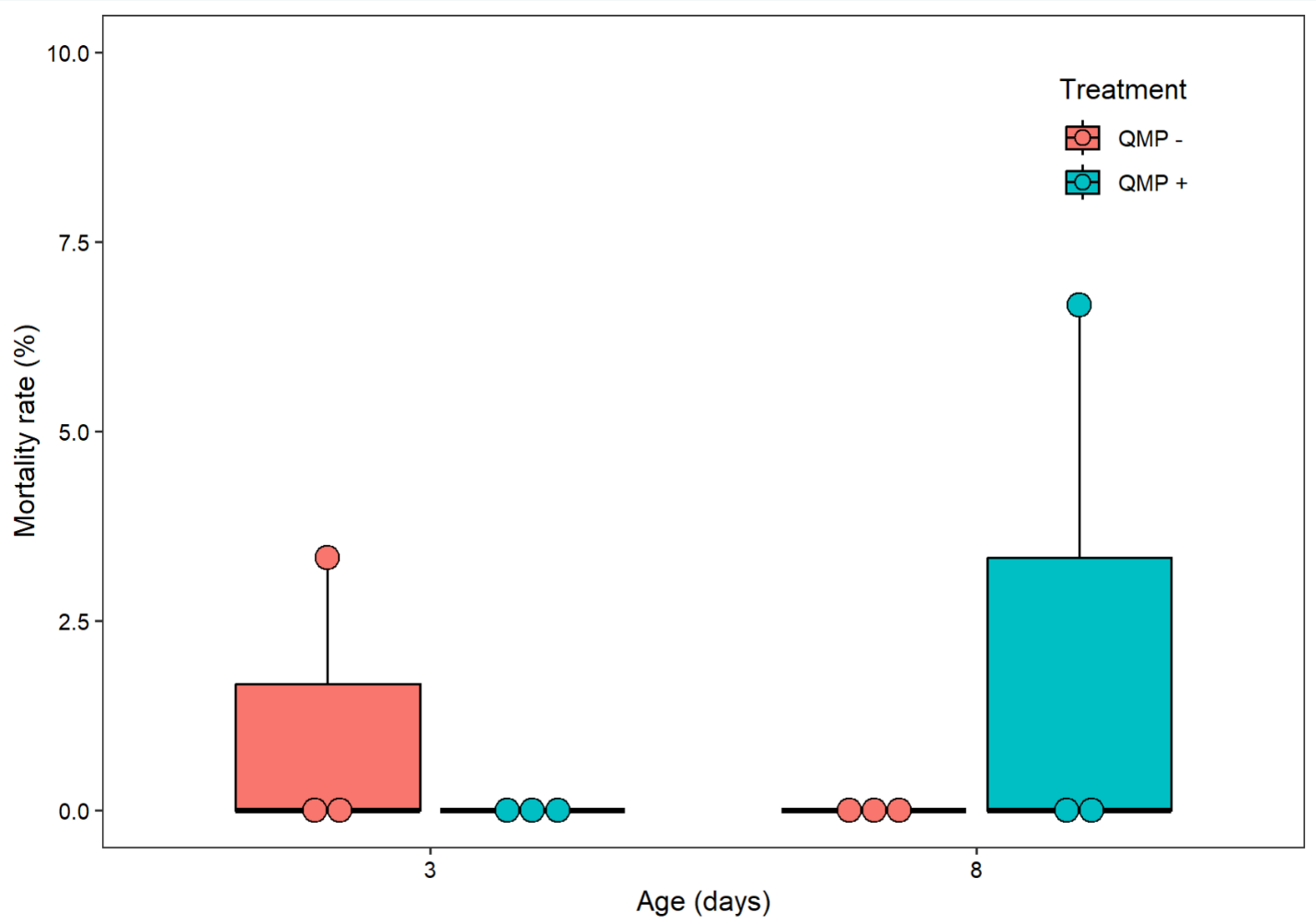
**

**S1 Fig. The relationship between age/QMP treatment and mortality of bees in %.** Morality rate did not significantly differ between the four treatment groups **(**Kolmgorov-Smirnov, *D*(10) = 0.167, *P* = 1).
