# Supplementary material for: The effects of queen mandibular pheromone on nurse-aged honey bee (Apis mellifera) hypopharyngeal gland size and lipid metabolism
Source: PLoS One. 2024 Sep 6;19(9):e0292500. doi: 10.1371/journal.pone.0292500 (PMC11379314; doi:10.1371/journal.pone.0292500)
Supplement: S2 Fig — (DOCX) [file pone.0292500.s002.docx]

**
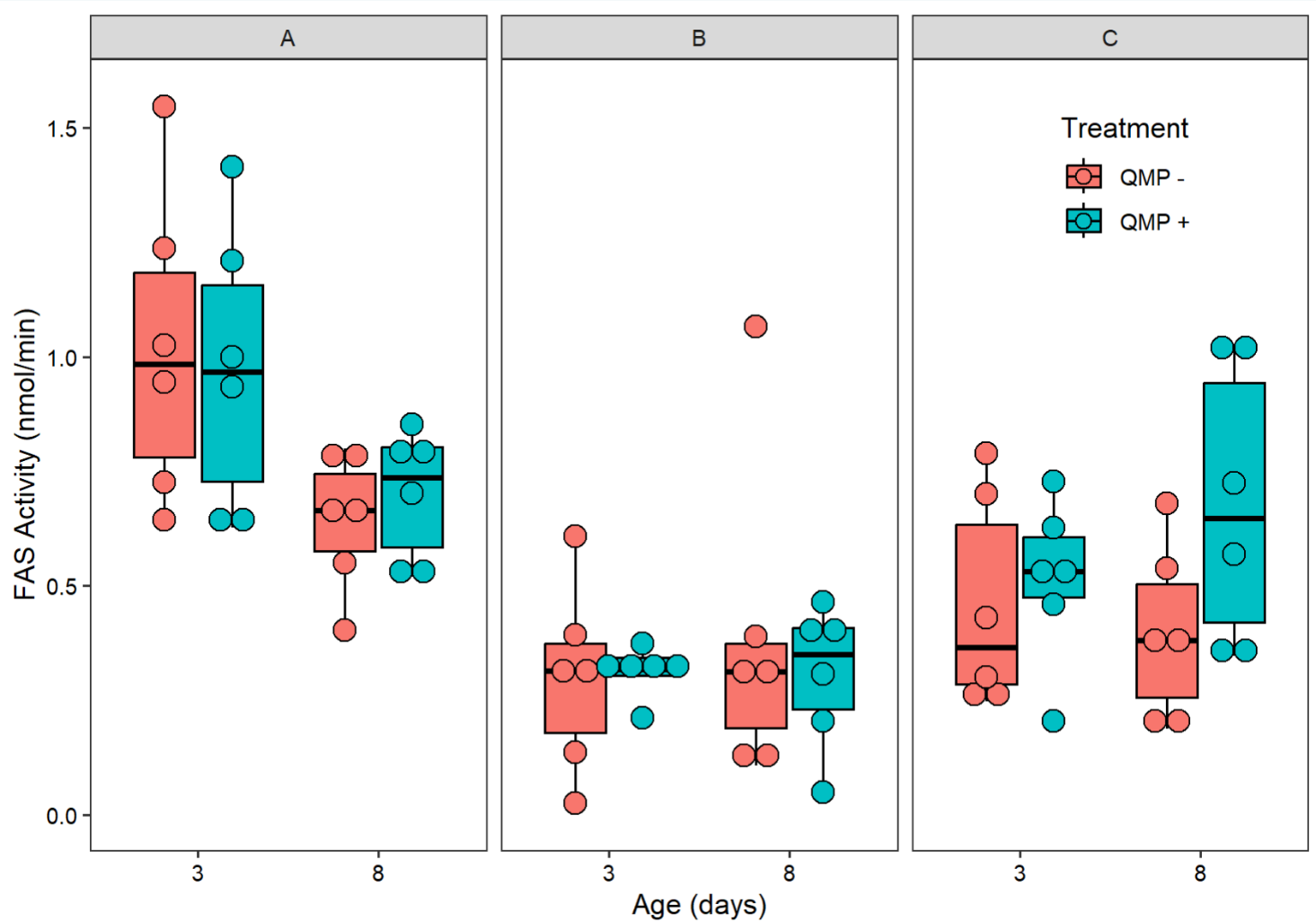
**

**S2A Fig. The effect of replicates on abdominal FAS activity measured in nmol/min/abdomen.**  Abdominal FAS activity significantly differed between the three replicates (F_2,60_ = 29.323, *P* < 0.001)

**
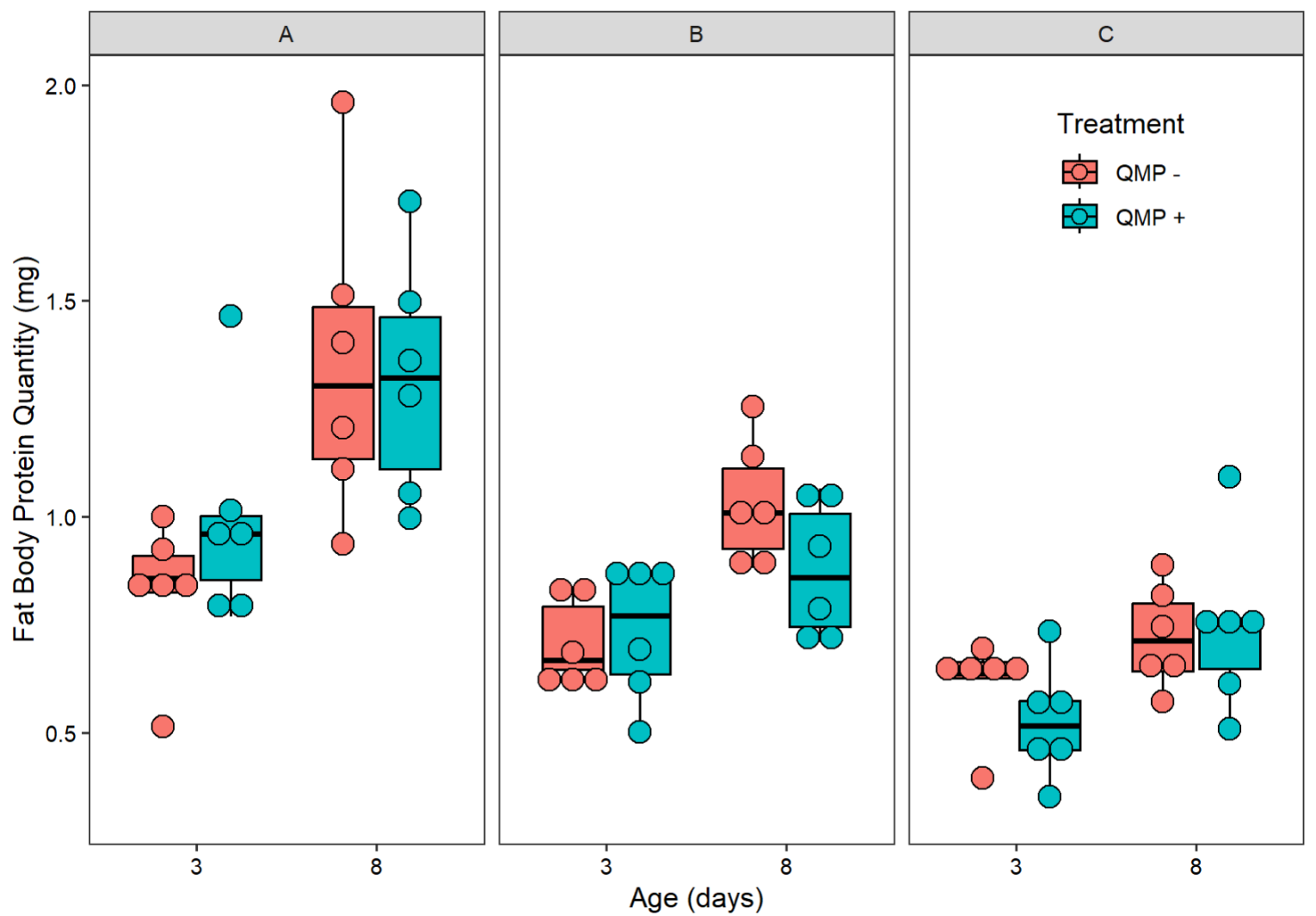
**

**S2B Fig. The effect of replicates on abdominal protein measured in mg.** Abdominal protein significantly differed between the three replicates (*F*_2,60_ = 36, *P* < 0.001).


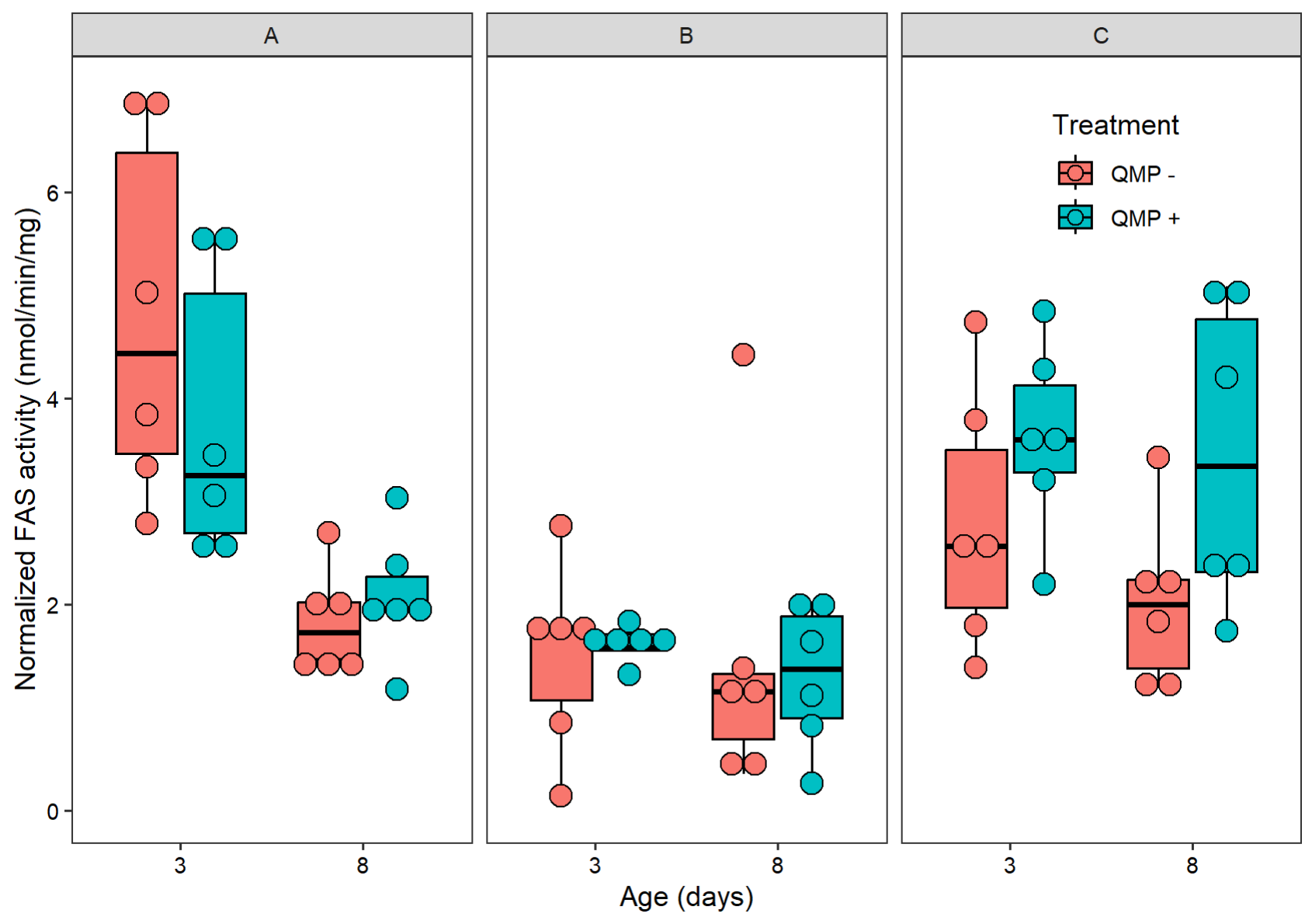


**S2C Fig. The effect of replicates on normalized FAS activity measured in nmol/min/mg.** Normalized FAS activity significantly differed between the three replicates (chi-squared = 23.87, *P* < 0.001, df = 2).

**
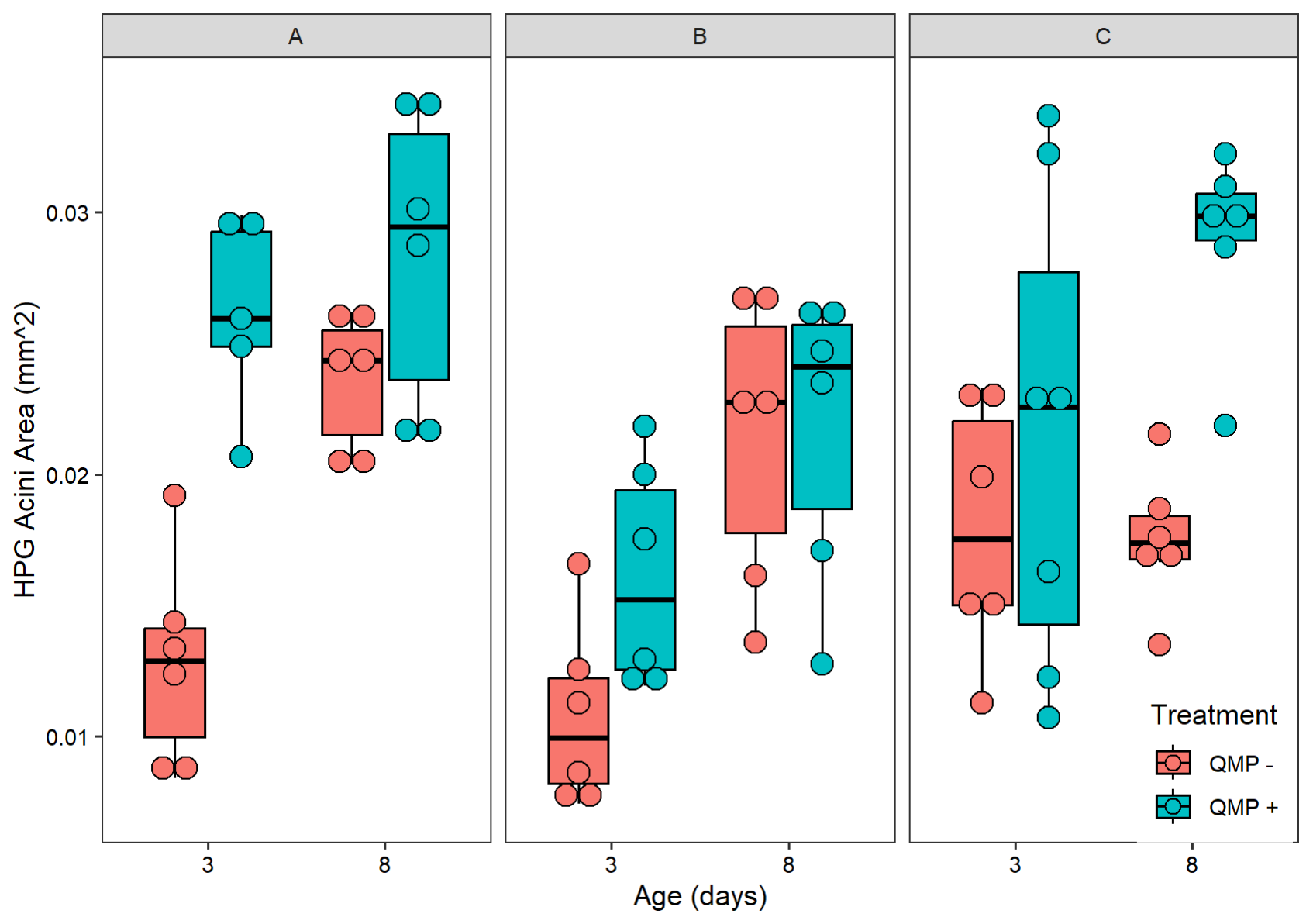
**

**S2D Fig. The effect of replicates on HPG acini area measured in mm^2^.** Mean HPG acini area significantly differed between the three replicates (*F*_2,60_ = 6.853, *P* = 0.00209).
