# Supplementary material for: The effects of queen mandibular pheromone on nurse-aged honey bee (Apis mellifera) hypopharyngeal gland size and lipid metabolism
Source: PLoS One. 2024 Sep 6;19(9):e0292500. doi: 10.1371/journal.pone.0292500 (PMC11379314; doi:10.1371/journal.pone.0292500)
Supplement: S1 File — (DOCX) [file pone.0292500.s003.docx]

**S1 File. The full regression plot analysis statistics between fat body protein and HPG acini area, abdominal FAS and HPG acini area, and fat body protein and abdominal FAS.**

**Fig 9. Regression scatter plot between fat body protein (mg) and HPG acini area (mm^2^).** The regression analysis generated a formula of mg = 0.010486 + 0.011270mm^2^. The standard error for HPG acini area was 0.002755 with a t value of 4.091. The standard error for fat body protein was 0.002947 with a t value of 3.558. The residual standard error was 0.005591 with df = 33. The fat body protein predictor variable had a significant *P* value of 0.00116 while the HPG acini area response variable had a significant *P* value of < 0.001. Each point represents the correlation between the pooled sample’s (2 bees per) fat body protein and HPG acini area (*N* = 35).

**Fig 10. Regression scatter plot between abdominal FAS (nmol NADPH oxidized/min) and HPG acini area (mm^2^).** The regression analysis generated a formula of (nmol/min) = 0.008093 + 0.016687mm^2^. The standard error for HPG acini area was 0.001928 with a t value of 8.654. The standard error for abdominal FAS was 0.003480 with a t value of 2.326. The residual standard error was 0.006096 with df = 33. The abdominal FAS predictor variable had a significant *P* value of 0.0158. The HPG acini area response variable had a significant *P* value of < 0.001. Each point represents the correlation between the pooled sample’s (2 bees per) raw FAS activity and HPG acini area (*N* = 35).

**Fig 11. Non-parametric linear regression scatter plot between abdominal protein (mg) and abdominal FAS (nmol oxidized NADPH/min).** The non-parametric Kendall–Theil Sen Siegel regression analysis generated a formula of mg = 1.5049 + 0.6252(nmol/min). The median absolute deviation for abdominal FAS was 1.2966 with a V value of 1951. The median absolute deviation for fat body protein was 1.4016 with a V value of 2265. The residual standard error was 1.186 with df = 70 and Efron’s pseudo r-squared was 0.0498. The fat body protein predictor variable had a significant *P* value of < 0.001. The abdominal FAS response variable had a significant *P* value of < 0.001. Each point represents the correlation between the pooled sample’s (2 bees per) fat body protein and abdominal FAS activity (*N* = 72).
