# Supplementary material for: The effects of queen mandibular pheromone on nurse-aged honey bee (Apis mellifera) hypopharyngeal gland size and lipid metabolism
Source: PLoS One. 2024 Sep 6;19(9):e0292500. doi: 10.1371/journal.pone.0292500 (PMC11379314; doi:10.1371/journal.pone.0292500)
Supplement: Attachment — Submitted filename: Response_to_reviewers (1).docx [file pone.0292500.s004.docx]

Dear reviewer/editor,

Thank you for the feedback on our submission to PLOS ONE and thank you for the opportunity to submit a revised version of our manuscript. We were pleased to receive thoughtful comments that are easy to address. Down below we have responded to each of your concerns with details on the revisions carried out on our manuscript since its original submission.

1. Reviewer comment: “*While the authors controlled important experimental variables, several important factors are missing in this experiment … [these] significantly affect the nutritional conditions of the bees under study and potentially the results obtained*.”

Authors’ reply: Cage experiments that eliminate these factors are standard practice in the field, specifically when investigating the role of pheromones and diet on physiological parameters, please see examples: [[1]–[3]](https://www.zotero.org/google-docs/?hMnPsN). We will provide this information in a new sentence on line 116: “To eliminate these issues and provide greater insight into specific nutrients in pollen, a number of recent studies have used artificial diets [16, 23, 24].”

2. Reviewer comment: “*Thirty bees per cage and three replicates per treatment is a modest experimental size for this type of study*.”

Authors’ reply: While we realize there is discussion about the appropriate sample size for consumption metrics, the number of bees we used is standard for inferences on physiological relationships [[3]–[5]](https://www.zotero.org/google-docs/?WUaWQa). We will explain these facts by inserting a clarifying sentence on line 119: “Cage experiments using cohorts of approximately 30 bees per cage maintained in an incubator are standard practice when investigating the role of pheromones and diet on physiological parameters and have been used in a number of different studies [16, 21, 25].”

3. Reviewer comment: “*Additional controls such as other diets (e.g., pollen) could have benefited the study*.”

Authors’ reply: Many articles address roles of dietary pollen, e.g. [[6]](https://www.zotero.org/google-docs/?5Yr337). Our goal was not to re-do these studies. Instead, we sought to optimize data reliability: Pollen is a natural compound that is highly variable and difficult to standardize. Our experiment eliminates this variability, thus allowing for greater reliability of data. We will clarify this context by inserting a sentence on line 113: “We chose to use artificial diets because many studies have shown strong effects of dietary pollen on worker physiology [11], but pollen composition is highly variable and difficult to standardize [21] and pollen may be contaminated with pesticides [22].”

4. Reviewer comment: “*What was the mortality rate of the bees under the different treatments? Was this significant?”*

Authors’ reply: Mortality was less than 7% over the course of the experiment. This is within the accepted range for honey bee cage studies [[7]](https://www.zotero.org/google-docs/?8T1Pot). This information was added on line 212: “Mortality rates were low (less than 7%) and within accepted ranges for cage experiments [19] for all ages and did not differ by QMP treatment (Kolmgorov-Smirnov, *D*(10) = 0.167, *P* = 1; Fig S1).” A new supplemental figure was added to the manuscript as well.

Thank you for reviewing our revisions. We would be pleased to assist you with securing additional reviewers for the manuscript if needed,

On behalf of the author team:

Angela Oreshkova (12/11/2023)

[[1]	D. Stabler, M. Al-Esawy, J. A. Chennells, G. Perri, A. Robinson, and G. A. Wright, “Regulation of dietary intake of protein and lipid by nurse-age adult worker honeybees,” *J. Exp. Biol.*, p. jeb.230615, Jan. 2020, doi: 10.1242/jeb.230615.](https://www.zotero.org/google-docs/?JPotsw)

[[2]	A. M. Trawinski and S. E. Fahrbach, “Queen mandibular pheromone modulates hemolymph ecdysteroid titers in adult Apis mellifera workers,” *Apidologie*, vol. 49, no. 3, pp. 346–358, Jun. 2018, doi: 10.1007/s13592-018-0562-6.](https://www.zotero.org/google-docs/?JPotsw)

[[3]	P. Fischer and C. M. Grozinger, “Pheromonal regulation of starvation resistance in honey bee workers (Apis mellifera),” *Naturwissenschaften*, vol. 95, no. 8, pp. 723–729, Aug. 2008, doi: 10.1007/s00114-008-0378-8.](https://www.zotero.org/google-docs/?JPotsw)

[[4]	E. Omar, A. A. Abd-Ella, M. M. Khodairy, R. Moosbeckhofer, K. Crailsheim, and R. Brodschneider, “Influence of different pollen diets on the development of hypopharyngeal glands and size of acid gland sacs in caged honey bees (Apis mellifera),” *Apidologie*, vol. 48, no. 4, pp. 425–436, Jul. 2017, doi: 10.1007/s13592-016-0487-x.](https://www.zotero.org/google-docs/?JPotsw)

[[5]	C. M. Grozinger and G. E. Robinson, “Endocrine modulation of a pheromone-responsive gene in the honey bee brain,” *J. Comp. Physiol. A Neuroethol. Sens. Neural. Behav. Physiol.*, vol. 193, no. 4, pp. 461–470, Mar. 2007, doi: 10.1007/s00359-006-0202-x.](https://www.zotero.org/google-docs/?JPotsw)

[[6]	V. Corby-Harris *et al.*, “Diet and pheromones interact to shape honey bee (Apis mellifera) worker physiology,” *J. Insect Physiol.*, vol. 143, no. October, p. 104442, 2022, doi: 10.1016/j.jinsphys.2022.104442.](https://www.zotero.org/google-docs/?JPotsw)

[[7]	S. K. Huang *et al.*, “Evaluation of cage designs and feeding regimes for honey bee (Hymenoptera: Apidae) laboratory experiments,” *J. Econ. Entomol.*, vol. 107, no. 1, pp. 54–62, Feb. 2014, doi: 10.1603/EC13213.](https://www.zotero.org/google-docs/?JPotsw)
