# Supplementary material for: The effects of queen mandibular pheromone on nurse-aged honey bee (Apis mellifera) hypopharyngeal gland size and lipid metabolism
Source: PLoS One. 2024 Sep 6;19(9):e0292500. doi: 10.1371/journal.pone.0292500 (PMC11379314; doi:10.1371/journal.pone.0292500)
Supplement: Attachment — Submitted filename: PLoS ONE QMP and HPGs Review.docx [file pone.0292500.s005.docx]

In this study, the authors measure how queen mandibular pheromone (QMP) affects important traits associated with nurse behavior in honey bees. They find that QMP increases hypopharangeal gland (HPG) size, even when bees are provided a lipid-deficient diet. Moreover, they find a significant positive correlation between HPG size and abdominal protein content. These effects of QMP are not explained by changes to lipogenic capacity. The researchers thoroughly interrogate the relationship between QMP and its affects on abdominal protein and HPG size. This research provides important steps forward in our understanding of how QMP modulates nurse traits. Please see my comments below on how to improve the reporting of this study.

Major Comments:

Sample size: First, throughout the results, sample sizes are not included in summary statistics. Please include whether experimental units are cages or bees and provide sample sizes in the summary statistics for how many bees of each treatment were used.

This experiment has an n=3 cages per treatment. This is fairly low but could perhaps be justified with clear explanation in the methods. Since the experimental unit for many of the results is bee, not cage, the authors could justify this design by making it clear that individual bees were the experimental unit, even though many other honey bee cage studies designate cage as the experimental unit. The authors should make this clear and provide proper explanation and justification. Yet, some of the results described in this paper are cage-level results. These results (food consumption, mortality) are not the central findings of the paper, but they are still discussed. In the cases of these results, there were no significant differences between treatments. However, a lack of significance could come from the very low sample size, and is not necessarily evidence that these metrics would not differ between treatments if the sample sizes were larger. This should be mentioned in the discussion/interpretation. More importantly, the figures that summarize these results are misleading because they employ boxplots, which display a 5-point summary of only 3-point data (see comment below about figures 3 and 4). A more appropriate chart type should be used instead.

Minor Comments:

Introduction:

Line 82: Why does the “natural food composition make it difficult to tease apart how QMP acts to influence physiology”? It’s not clear what is meant by “natural food composition” or how it relates to QMP.

Line 85: Are there many possibilities? I suggest either outlining some more potential possibilities, or removing this final line of the paragraph.

Line 88: Can you please define lipogenic capacity here?

Line 95: Why does your hypothesis make predictions about HPG size? Can you better explain your reasoning for why you hypothesize that increased lipogenic capacity would mean larger HGPs?

Line 96: Why did you use these two different ages? Can you outline your hypothesis/predictions for 3-day vs. 8-day old bees?

Line 125 (Fig 1 legend): What cages are these dimensions for? Are they different from the Plexiglas cages described in line 109 of the methods? The dimesons are not the same as described there.

Methods:

Line 129: These sample sizes (3 cages per treatment) seems fairly low. Although the authors addressed a previous reviewer’s concerns about the number of bees per cage (line 120), can you also include justification for the small replicate size?

Line 199: The sample sizes were n=3. Does this mean 3 *per treatment*, or 3 total?

Line 202: What is the makeup of these sample sizes (18, 35, and 72)? Are they pooled within cages or across cages? How are cages and treatments represented within these sample sizes? Are they balanced? If not, please give specifics of how many bees were used from each treatment, per cage.

Results:

Line 206-214: Please include sample sizes for test statistics. Also, please explain “between replicates”: Are these stats explanatory for all 4 treatments?

Figures 3 and 4, and their legends:

- Include “(days)” in x-axis labels.

- These figure legends include the line “Outliers are points outside the maximum and minimum of the distribution.” These figures do not contain outliers (because n=3), so this line can be removed for each legend. Moreover, these legends include a description of the interquartile range, but no actual values represent the interquartile range (because n=3). I believe that boxplots are not the correct way to represent the data in these figures. A boxplot is used to summarize a larger dataset down to 5 points (min, max, median, and the two interquartiles). Because the actual data here contains *fewer* than 5 points, a boxplot is not a summary but an *extrapolation* of the data and gives the false impression of summarizing a larger dataset. Either a simple point plot or bar charts would be more honest visual representations of the data.

Line 233: “significant differences between the three replicates”: For which treatment? This needs to be clear each time a significant differences is reported between replicates (like in line 237, for example).

Line 247: Were pooled bees from the same cage? Were there 6 pools per cage?

Figures 5, 6, 7, and 8 legends: “Outliers are points outside the maximum and minimum distribution.” How do you have points outside the max and min of the distribution? Do mean these fall outside the expected variation of the data?

Fig 10 legend: Provide sample sizes in the summary statistics for how many bees of each treatment were used.

Fig 11 legend: Provide sample sizes in the summary statistics for how many bees of each treatment were used.

Table 1: Why did you not include normalized FAS activity in this matrix?

Discussion:

Line 328: Replicate effects may also be a result of the low cage sample size. I do not think they are irrelevant.

Line 358: How does this potentially explain the differing results? Did those other studies not use young workers?

Line 359: Change “the mechanism… is” to “the mechanism… could be”, since this is a speculation.

Line 373: I do not completely follow this logic. If QMP mobilizes abdominal proteins to the HPGs to increase HPG size, wouldn’t abdominal protein and HPG size be negatively correlated in QMP+ cages (since a finite amount of protein is being moved from abdomen to HPGs)?
