# Supplementary material for: The effects of queen mandibular pheromone on nurse-aged honey bee (Apis mellifera) hypopharyngeal gland size and lipid metabolism
Source: PLoS One. 2024 Sep 6;19(9):e0292500. doi: 10.1371/journal.pone.0292500 (PMC11379314; doi:10.1371/journal.pone.0292500)
Supplement: Attachment — Submitted filename: Response to Reviewers.docx [file pone.0292500.s006.docx]

Dear reviewers/editor,

Thank you for the feedback on our submission to PLOS ONE and thank you for the opportunity to submit a revised version of our manuscript. We were pleased to receive thoughtful comments that we are able to address. Down below we have responded to each of your concerns with details on the revisions carried out on our manuscript since its original submission (the line numbers refer to the track changes manuscript document):

1. Reviewer #1 comment: *“… The authors do a good job discussing the implications of their small sample size on the results of their diet consumption analyses, but might this have implications for other aspects of their study? If the authors don’t think so, please add some explanation as to why this was unlikely to have been an issue to the discussion. … It would have been interesting to see how FAS activity differed depending on nutrient availability… Finally, the authors looked at HPG size but not content. Could protein/lipid composition vary in a way that is related to FAS activity?”*

Authors’ reply: We understand that our original submission was unclear in its communication of sample sizes. In contrast to the small sample size for the consumption results, we had a large sample size for the other components of the study, for example 72 total samples for the FAS activity. We added discussion of sample size to L428. We agree that the effects of dietary nutrition on FAS activity are worth exploring though outside the scope of this study. We have addressed this in a separate study and added a statement and citation explaining this on L422. We added a statement addressing the limitation of our study in not measuring FAS content or secretory activity on L485.

2. Reviewer #1 comment: *“HPGs secrete worker jelly and royal jelly, which are different. This should be adjusted in your abstract and when it is mentioned again in the text. Likewise, large HPGs do not always signify nurse bee-like physiology. Winter bees also have enlarged HPGs.”*

Authors’ reply: It is true that the composition of worker and royal jelly are different, as noted in the reference provided by the reviewer. To avoid confusion, we replaced “royal jelly” by the term “proteinaceous jelly” in lines 35, 36, and 79 as both types of jelly contain protein as the primary macronutrient [1]. To further take action on this important comment, we added a new sentence on line 474: “It is known that HPGs contain a high concentration of lipids [15]. This, combined with nurses having high fat body lipids relative to foragers, suggests that HPG lipids are important for jelly production.”

3. Reviewer #1 comment: *“L134: What dose (Qeq’s) does this synthetic lure represent?...The authors do acknowledge that the use of synthetic QMP may have affected their results, but could dose have played a role?”*

Authors’ reply: We understand that our original submission was unclear in what dosage of synthetic lure we used and how the dosage could have played a role in our study. Thus, we added details on Qeqs to L157 and justification of the application and dose to L158.

4. Reviewer #1 comment: *“L175: Starting sentences with a number should be avoided.”*

Authors’ reply: We agree that starting sentences with a number should be avoided, thus, we corrected line 195 to now read: “Per treatment group, 18 heads were dissected, resulting in a total of 72 heads.”

5. Reviewer #1 comment: *“When Kruskal-Wallis tests were used, did you only compare QMP treatment within age groups and age groups within QMP treatment?”*

Authors’ reply: We used the Kruskal-Wallis test to compare all four treatment groups (combination of QMP and age) rather than comparing QMP and age separately. This was clarified on line 216.

6. Reviewer #1 comment: *“L227 … Consider eliminating the unnormalized data from this ms.”*

Authors’ reply: While we agree that eliminating redundant figures is important, there are two important reasons to keep the unnormalized data: first is that the normalization factor itself is significantly different between age groups, potentially confounding interpretation. For full transparency, we want to show the unnormalized trend here. Second is that the unnormalized data are themselves meaningful, since we are reporting the total lipogenic capacity in two bee abdomens. Reporting these data (such as total lipids per abdomen) are standard practice in honey bee research and useful for aiding in interpretation of the data.

7. Reviewer #1 comment: *“I’m not sure that the authors fully examined whether workers increase HPG size through increased food consumption. They only used a suboptimal, low lipid diet. It’s possible that consumption rates and FAS activity might differ depending on diet composition and QMP presence.”*

Authors’ reply: We agree that consumption rates differ depending on diet composition and QMP presence as highlighted in two studies mentioned in our manuscript [2, 3]. We further explained the limitations of our experiment by inserting the following statements on line 420: “Furthermore, our study used a lipid deficient diet while Ament and colleagues [13] and Peters and colleagues [14] used a combination of diets consisting of only pollen, pollen and sucrose, or only sucrose. There may be some interacting effects between QMP and nutrition which we did not test for.”

8. Reviewer #1 comment: *“It seems far more likely that there is some interaction between diet and QMP that isn’t captured by this experiment.”*

Authors’ reply: We agree that there are limitations to our experiment as we only studied one type of diet. We inserted a statement on line 441 to explain how diet (among other factors) may contribute to the differing results of QMP affecting abdominal lipid stores: “If the activity levels and lipid catabolism rates are different between our bees and the bees used in the other studies [11,12,13] due to varying ages, environment, and diet, this could explain the inconsistency of whether QMP affects abdominal lipid stores.”

9. Reviewer #1 comment: *“I’m not really seeing the connection between this statement and reference 20.”*

Authors’ reply: We apologize that the incorrect reference was cited in the original submission. The correct reference to the statement on line 472 is reference 44. However, we have decided to remove this statement from the revised manuscript as we felt it did not provide relevant information to the passage.

10. Reviewer #2 comment: *“Please include whether experimental units are cages or bees and provide sample sizes in the summary statistics for how many bees of each treatment were used. This experiment has an n=3 cages per treatment. This is fairly low but could perhaps be justified with clear explanation in the methods … The authors should make this clear and provide proper explanation and justification. Yet, some of the results described in this paper are cage-level results.”*

Authors’ reply: We understand that our original submission was unclear of what experimental units we used. We rewrote the passage on line 228 to clarify our experimental units and provide justification to our low consumption metric sample size: “For all measurements except for consumption, the experimental unit was bees or pooled bees. For consumption metrics, the cage was the experimental unit. A balanced study design was employed for all factors. The sample size for cage level consumption metrics was low at *n* = 3 cages per treatment as this was not the focus of our study and the metrics describe the physiological parameters of the bees.”

11. Reviewer #2 comment: *“These results (food consumption, mortality) are not the central findings of the paper, but they are still discussed … However, a lack of significance could come from the very low sample size, and is not necessarily evidence that these metrics would not differ between treatments if the sample sizes were larger. This should be mentioned in the discussion/interpretation.”*

Authors’ reply: To clarify that a lack of significance between protein/sucrose consumption among the four treatment groups may be due to our low sample size, we revised line 417 to now read: “The lack of significant differences in food consumption between our treatments could be due to our small sample size. Thus, Because our study had the smallest sample size, our data should be interpreted cautiously.”

12. Reviewer #2 comment: *“A more appropriate chart type should be used instead [for food consumption data].”*

Authors’ reply: We agree that a more appropriate chart type should be used as the sample size for food consumption data was below 5. Thus, we revised figures 3 and 4 to now be dot plots rather than box plots. Please refer to the new attached figures for these changes.

13. Reviewer #2 comment: *“Why does the ‘natural food composition make it difficult to tease apart how QMP acts to influence physiology’? It’s not clear what is meant by “natural food composition” or how it relates to QMP.”*

Authors’ reply: We understand that we were not clear what ‘natural food consumption’ means. We define natural food consumption as the “variable macronutrient composition (lipids and amino acids) of pollen as well as the potential contamination of pesticides” and this was added to line 83. Furthermore, we clarified how consumption relates to QMP with a reference added to line 81: “Furthermore, there has been research that exposure to QMP increases both pollen and sucrose solution consumption in nurse-aged bees [13].”

14. Reviewer #2 comment: *“Line 85: Are there many possibilities? I suggest either outlining some more potential possibilities, or removing this final line of the paragraph.”*

Authors’ reply: We understand that ending this passage openly is vague and can confuse the reader of whether there are more possibilities outside of what we mentioned. Therefore, the final sentence of line 89 was removed for concision and we ended the paragraph on line 87 with our question of: “Are the QMP-induced increases in lipid stores and HPG size behaviorally modulated by increasing consumption of lipid-containing pollen, or are they due to effects on metabolic pathways regulating lipid and protein synthesis and storage?”

15. Reviewer #2 comment: “*Can you please define lipogenic capacity here?*

Authors’ reply: We understand that we did not define lipogenic capacity well in our original submission and thus added a brief definition of lipogenic capacity to L96.

16. Reviewer #2 comment: *“Why does your hypothesis make predictions about HPG size? Can you better explain your reasoning for why you hypothesize that increased lipogenic capacity would mean larger HGPs?*

Authors’ reply: We understand that our hypothesis reasoning for why increased lipogenic capacity would mean larger HPGs could be explained better. We added additional explanations and citations to L99-105.

17. Reviewer #2 comment: *“Why did you use these two different ages? Can you outline your hypothesis/predictions for 3-day vs. 8-day old bees?”*

Authors’ reply: We added an explanation of our reasoning for using two age groups on L115.

18. Reviewer #2 comment: *“(Fig 1 legend): What cages are these dimensions for? Are they different from the Plexiglas cages described in line 109 of the methods?”*

Authors’ reply: We apologize for the confusion in cage dimensions. We remeasured and inputted the correct cage dimensions on line 128 and 145 which now read: “The dimensions measured were 16 cm × 12 cm × 9 cm. Because approximately half the cage was used, the effective depth was 6 cm.”

19. Reviewer #2 comment: *“These sample sizes (3 cages per treatment) seem fairly low. Although the authors addressed a previous reviewer’s concerns about the number of bees per cage (line 120), can you also include justification for the small replicate size?”*

Authors’ reply: We understand that our original submission did not discuss the justification for our replicate size. We added justification of the level of replication to L150.

20. Reviewer #2 comment: *“The sample sizes were n=3. Does this mean 3 per treatment, or 3 total? What is the makeup of these sample sizes (18, 35, and 72)? Are they pooled within cages or across cages? How are cages and treatments represented within these sample sizes? Are they balanced? If not, please give specifics of how many bees were used from each treatment, per cage.”*

Authors’ reply: We understand that the sample size section in our original submission was not adequately explained. We rewrote the passage beginning on line 228 to clarify the makeup of our sample sizes and whether they are balanced: “For all measurements except for consumption, the experimental unit was bees or pooled bees. For consumption metrics, the cage was the experimental unit. A balanced study design was employed for all factors. The sample size for cage level consumption metrics was low at *n* = 3 cages per treatment as this was not the focus of our study and the metrics describe the physiological parameters of the bees. For measuring abdominal FAS activity, normalized FAS activity, abdominal protein, and HPG acini area, 2 bees were pooled for each biological sample and 6 samples totaling 12 bees were collected from each cage for all four treatment conditions. This totaled 72 biological samples pooled from 144 abdomens as three replicates were performed. Thus, the sample size was *n* = 18 pooled samples per treatment group for these three parameters. The sample size for the regression analyses was *n* = 35 pooled samples as there was a dissection issue for one of the bees and only half of the HPGs of the 24 pooled samples per replicate were measured, except for the case of abdominal protein and abdominal FAS which was *n* = 72 pooled samples (144 total abdomens).”

21. Reviewer #2 comment: *“Line 206-214: Please include sample sizes for test statistics. Also, please explain ‘between replicates’: Are these stats explanatory for all 4 treatments?” “Line 233: ‘significant differences between the three replicates’: For which treatment? This needs to be clear each time a significant difference is reported between replicates (like in line 237, for example).”*

Authors’ reply: We included sample sizes for food and sucrose consumption on lines 245 and 247. We apologize for not outlining our replicate effects clearly in the original manuscript. To address the reviewers' comments to lines 206 - 214 and line 233, we have included a detailed explanation of what the replicate effect is beginning on line 219: “For each parameter analyzed with either a parametric or non-parametric test, replicate effects were tested for. These describe whether there were significant differences between each replicate and if there was an independent effect of doing a replication of the experiment overall.”

22. Reviewer #2 comment: *“Figures 3 and 4, and their legends: include ‘(days)’ in x-axis labels; These figure legends … Either a simple point plot or bar charts would be more honest visual representations of the data.”*

Authors’ reply: We included ‘days’ in the x-axis labels for figures 3 and 4 (lines 253 and 259). We agree that a more appropriate chart type should be used as the sample size for food consumption data was below 5. Thus, we revised figures 3 and 4 to now be dot plots rather than box plots. Please refer to the new attached figures for these changes.

23. Reviewer #2 comment: *“Were pooled bees from the same cage? Were there 6 pools per cage? Figures 5, 6, 7, and 8 legends: ‘Outliers are points outside the maximum and minimum distribution.’] How do you have points outside the max and min of the distribution? Do these fall outside the expected variation of the data?”*

Authors’ reply: We agree that sample sizes should better be explained for figure 5,6,7, and 8 legends. We clarified how the sample size was calculated for these figures with the following sentence added to lines 285, 298, 321, and 343: “As there were 6 pools per cage (12 abdomens/bees sampled) and three replicates performed, the sample size is *n* = 18 pooled samples.” We amended the figure legends to explain that whiskers are calculated as 1.5 times the interquartile range and outliers are data points outside the whiskers that depict the expected variation of the data.

24. Reviewer #2 comment: *“Fig 10/11 legend: Provide sample sizes in the summary statistics for how many bees of each treatment were used.”*

Authors’ reply: We agree that sample sizes should better be explained for figure 9, 10 and 11 legends. We included how many bees of each treatment were used in the figure 9, 10, and 11 legends on lines 363, 371, and 379. In the case of the figure 9 and 10 legends, we added the following clarification: “Each point represents the correlation between the pooled sample’s (2 bees per) abdominal protein and HPG acini area (*n* = 35 pooled bees). 18 bees per treatment were used except for the case of the 3-day-old QMP + treatment group in the first replicate as there was a dissection issue. An additional sample was measured but not included in the regression analysis.” In the case of the figure 11 legend, we added the following clarification: “36 bees per treatment were used, resulting in *n* = 72 pooled bees and a total of 144 abdomens.”

25. Reviewer #2 comment: *“Table 1: Why did you not include normalized FAS activity in this matrix?”*

Authors’ reply: We apologize if there was any confusion on why normalized FAS activity was not included in the table. We provided a justification for why we did not include it in the table legend on line 386: “Normalized FAS activity was not included in this matrix as it was used as a summary statistic and will co-vary from the factors that it was calculated from (abdominal protein/abdominal FAS activity).”

26. Reviewer #2 comment: *“Replicate effects may also be a result of the low cage sample size. I do not think they are irrelevant.”*

Authors’ reply: While it is meaningful to mention potential sources of replicate differences within the manuscript, our goal was not to focus on these results. However, we acknowledged the effect of low cage sample size on replicate effects beginning on line 392: “In interpreting our results, it is important to note that there were several significant differences between experimental replicates. In the case of the sucrose consumption data, this is likely due to our small sample size. There were also significant replicate effects within the abdominal FAS activity data, abdominal protein data, and average HPG acini data, where we had relatively large sample sizes (*n* = 18). Since we sourced bees in the experiment from three different colonies, these replicate effects may have been a result of biological variation among bees with different genetic backgrounds, but we cannot rule out contributions of technical variation as well.”

27. Reviewer #2 comment: *“How does this potentially explain the differing results? Did those other studies not use young workers?”*

Authors’ reply: The other studies cited did use young bees and this was clarified on lines 436 and 438. To further explain the diversity of results of whether QMP affects abdominal lipid stores, we inserted new insight beginning on line 439: “The mechanism underlying the larger lipid stores could be reduced energy expenditure by decreasing the catabolism rate of lipids in young bees. Future work could look at the lipid breakdown rate in bees exposed or not exposed to QMP. If the activity levels and lipid catabolism rates are different between our bees and the bees used in the other studies [11,12,13], this could explain the inconsistency of whether QMP affects abdominal lipid stores.”

28. Reviewer #2 comment: *“Change “the mechanism… is” to “the mechanism… could be”, since this is a speculation.”*

Authors’ reply: We changed line 439 to reflect this comment as we agree on the reviewer’s reasoning: “The mechanism underlying the larger lipid stores could be reduced energy expenditure by decreasing the catabolism rate of lipids in young bees.”

29. Reviewer #2 comment: *“If QMP mobilizes abdominal proteins to the HPGs to increase HPG size, wouldn’t abdominal protein and HPG size be negatively correlated in QMP+ cages (since a finite amount of protein is being moved from abdomen to HPGs)?”*

Authors’ reply: We recognize the argument that there may be a negative correlation between abdominal protein and HPG size if a fixed amount of protein from the abdomen is being transferred to the HPGs (assuming our bees are within a closed system). To address this concern, we revised our manuscript to focus on the change of Vg synthesis rather than the mobilization of the protein. We also added further insight on the juvenile hormone (JH) effect on HPG development. These changes were made on line 451 and 461 respectively.

[1] Yang W, Tian Y, Han M, Miao X. Longevity extension of worker honey bees (*Apis mellifera*) by royal jelly: optimal dose and active ingredient. PeerJ. 2017; 5(3118). Pmid: 28367370

[2] Ament SA, Chan QW, Wheeler MM, Nixon SE, Johnson SP, Rodriguez-Zas SL, et. al. Mechanisms of stable lipid loss in a social insect. Journal of Experimental Biology. 2011;214(22): 3808 - 3821. Pmid:22031746

[3] Peters L, Zhu-Salzman K, Pankiw T. Effect of primer pheromones and pollen diet on the food producing glands of worker honey bees (*Apis mellifera L.*). Journal of Insect Physiology. 2010;56(2): 132 - 137. Pmid:19799907
