# Supplementary material for: The effects of queen mandibular pheromone on nurse-aged honey bee (Apis mellifera) hypopharyngeal gland size and lipid metabolism
Source: PLoS One. 2024 Sep 6;19(9):e0292500. doi: 10.1371/journal.pone.0292500 (PMC11379314; doi:10.1371/journal.pone.0292500)
Supplement: Attachment — Submitted filename: Response to Reviewers (6.28.24).docx [file pone.0292500.s007.docx]

Dear reviewers/editor,

Thank you for the additional feedback on our submission to PLOS ONE and thank you for the opportunity to submit a second revised version of our manuscript. We were pleased to receive additional thoughtful comments that we are able to address. Down below we have responded to each of your new concerns with details on the revisions carried out on our manuscript since its original submission (the line numbers refer to the new track changes manuscript document):

1. Reviewer #2 comment: *“Please represent significant differences somehow in your figures.”*

Authors’ reply: We agree that significant differences should be represented in our figures. Please refer to attached figures 6, 7, and 8 to view these changes. Furthermore, we adjusted the legends of these figures (L310, L330 and L351) to make note of the symbols we used in the figures and what they signify.

2. Reviewer #2 comment: *“Standard practice for analyzing FAS activity is to normalize it to protein content largely because protein content will vary between samples. You may be seeing a relationship between FAS activity and abdominal protein content because FAS is partially dependent on amino acids and carbohydrates as substrates. Why wouldn’t you depict it the way you analyzed it in your figures? And why isn’t it represented this way in your table?”*

Authors’ reply: We agree that standard practice for analyzing FAS activity is to normalize it to protein content. However, we did choose to report both normalized and non-normalized data and have added a clarification to L188 to explain why: “For a more complete picture of the metabolic state of the bee, we report total abdominal FAS activity as well as FAS activity normalized to mg of abdominal protein. Because our dissection approach collects the entire abdominal cuticle and attached fat body, it produces a metric of total lipogenic capacity per abdomen, similar to past studies that have measured total lipids per abdomen using the same dissection technique [7, 12, 15, 16]. To control for potential differences in protein extraction efficiency, we also measure protein concentration in each sample and normalize FAS activity relative to extracted protein. Comparing results from the two metrics makes it clearer when treatment group differences are due to changes in the quantity of activated FAS or due to changes in the normalization factor itself.” We apologize if we were not clear as to why we reported both data sets.

3. Reviewer #2 comment: *“N=18 is a reasonable sample size for FAS analysis but it’s small for HPGs. The authors continually state that 18 is a relatively large sample size. Relative to what?”*

Authors’ reply: We have removed the reference to large sample size on L398 and altered the wording on L425-426 to make it clear that our HPG sample size is large relative to our cage-level consumption data.

4. Reviewer #2 comment: *“I pointed out in my last review that it isn’t grammatically correct to begin a sentence with a number. There are several new instances of this error in this version that need to be addressed.”*

Authors’ reply: We apologize that there were several more instances of beginning our sentences with a number. We corrected this on the following lines: L118, L141, L223, L364, L372, and L379.

5. Reviewer #2 comment: *“L216-222: I'm still unclear on some aspects of the stats. I see that you used KW or anova depending on whether the data satisfied assumptions. How were replicate effects evaluated for KW models? Did you just run separate KW tests for each rep? For ANOVA models, was replicate treated as a factor? Did you do anything to account for possible interaction between factors for ANOVA and KW models? I imagine there were limitations associated with using the KW models since you can't run the equivalent of a 2-way anova. How did you try to address those?”*

Authors’ reply: We added information on L215 and L227 about how we addressed replicate effects and interactions between factors for ANOVA. We added two sentences on L229-L232 outlining our strategy for using a Kruskal-Wallis effect to test for replicate and treatment effects in the normalized FAS data.

6. Reviewer #2 comment: *“L233-238: All of this should perhaps be described earlier, when discussing FAS activity assay methods.”*

Authors’ reply: We agree that there should be a description of how 144 bee abdomens were sampled across three replicates in our FAS activity assay methods and apologize if there was any confusion surrounding this. We added a section on L182 to better describe our sampling methods: “This assay was replicated 3 times. In each replicate, 2 bees were pooled for each biological sample and 6 samples totaling 12 bees were collected from each cage for all four treatment conditions. This totaled with a total of 144 abdomens sampled across three replicates.”

7. Reviewer #3 comment: ‘*Thank you for addressing my comments. One final note, please add a definition of "queen equivalent" (line 157).’*

Authors’ reply: We agree that the term ‘queen equivalent’ should be defined and therefore, we clarified this on L156: “One queen equivalent represents the amount of QMP a mated queen will produce in a 24-hour period and contains 200 mg of ODA, 80 mg of 9-HAD, 20 mg of HOB, and 2 mg of HVA [34]”.
